# A framework for smart construction contracts using BIM and blockchain

**DOI:** 10.1038/s41598-023-37353-0

**Published:** 2023-06-23

**Authors:** Mohamed A. Kamel, Emad S. Bakhoum, Mohamed M. Marzouk

**Affiliations:** 1 Orascom Construction, Cairo, Egypt; 2 Civil Infrastructure Engineering and Management Department, Nile University, Giza, Egypt; 3 Civil Engineering Department, National Research Centre, Cairo, Egypt; 4 Construction Engineering and Management, Structural Engineering Department, Faculty of Engineering, Cairo University, Cairo, Egypt

**Keywords:** Civil engineering, Electrical and electronic engineering

## Abstract

Poor payment practices are perceived as one of the biggest challenges facing the construction industry. Since payments are issued according to project contract terms, the project’s cash flow is inherently affected by the contract and how parties fulfill their obligations. This research proposes a framework for payment automation in construction projects to achieve smart construction contracts. Payments are automatically issued upon satisfying contract conditions using blockchain. Cryptocurrency is proposed to be utilized in the framework to execute the contract terms with no need for a third party to process project payments. 5D BIM is used to model the geometry of buildings and visualize project progress together with payment status using Autodesk Revit, Navisworks, and Primavera P6. The developed framework has the potential to reduce the consequences of poor payments. An actual case study for a construction project in Cairo, Egypt is worked out to demonstrate the main features of the proposed framework. The results of the case study reveal that project cash flow is secured and payments are instantly issued. Moreover, electronic records of payments are kept on the blockchain.

## Introduction

In 2018, the construction industry’s contribution to the UK was in the order of £117 billion, forming 6% of the UK GDP^1^. While global construction spending is expected to jump from $10.4 trillion in 2017 to $12.4 trillion in 2022^2^. Due to the significance of the industry to the different global economic systems, researchers have considered its issues trying to solve them or provide better practices. Amongst the challenges facing the construction industry, poor payment practices are of most importance^3–5^. Although project payments are regulated in the contract, they can’t be forced or trusted to be paid directly when they become due^6^. Whenever the contractor is entitled to a sum of money in consideration of the achieved works, the payment of such money could be delayed, not paid at all, or withheld because of disputes between project parties. This disturbance to projects or corporates’ cashflow may turn into insolvency, and eventually business failure^3,5,7^. The research is addressing the cash flow problems resulting from poor payment practices and the problem of contract enforceability.

To save construction projects’ cash flow, the management of construction contracts needs to be improved. In order to efficiently manage a construction project, the project’s cashflow should be always secured. Payments can be secured and automated using blockchain and smart contracts. This approach can save the Contractor and the whole supply chain from insolvency out of late payments^4^, reduce the risks related to payments, or lack of, and prolonged payment procedures under traditional scheme^8^. Blockchain is known as an append-only, immutable ledger, in other words, the blockchain is unchangeable based on cryptographic hashing. Immutability is present in nearly all blockchains^9^. Blockchain solves the problem of trust between parties and eliminates the need for a central trusted third party^10^. The unique nature of blockchain supports the consideration of blockchains as trust machines that could be used in the construction industry^11,12^. The use of blockchain technology is sought as a solution that can reduce project disputes arising from delayed payments and bring down the risks associated with cash flow problem^13^.

The coding of contract clauses into a machine-readable format, also known as smart contracts, has been proposed by some researchers as a solution to the problems of poor payment practices and inefficient contract management^6^. Smart Contracts are defined as “a computer program that is self-verifying, automatically executable, and resists manipulation”^14^. The integration between smart contracts, blockchain, and BIM technologies is progressively affirmed in the construction industry reports and initiatives, however, theoretical grounds and empirical proofs-of-concept necessary for the widespread adoption of the above technologies are very limited^15^. The current literature offers no advanced engine that can automate contract clauses or simulate a specific scenario^16^.

BIM technology has a pivotal effect on the digital transformation of the construction industry as a means of integration between parties that defines their responsibilities and obligations^17,18^. The construction visualization is sought as the gap closing between the project’s different actors. This nature of BIM enhances collaboration between project parties, and hence builds trust between them.

Some researchers have successfully implemented the integration of Blockchain, BIM, and smart contracts for issuing payments to 7 subcontractors in real construction projects in the United States of America and Canada^19^. Abrishami and Elghaish^20^ developed a framework that utilizes 5D BIM, in addition to Blockchain and smart contracts technologies, to achieve automated payments based on achieving milestones.

The main objective of this paper is to enhance construction projects’ cash flow at the different levels of framework adoption, through a novel framework that utilizes smart contracts, blockchain, and BIM technologies. The output of this framework is a smart construction contract, which proposes a mechanism that supports integrity, and enables the contract enforceability. The proposed framework has potential savings on the cost of construction projects through reduction of administrative fees. Moreover, the research proposes the development of construction smart contracts framework to assist the management teams with payment-related clauses and cashflows’ enhancement, the automation of construction contract clauses as an effort towards smart contracts, the proposal of blockchain as an efficient alternative financial system to the traditional one used in the construction industry and the proof of potential savings out of using blockchains instead of traditional financial schemes incorporating banks as third parties.

## Literature review

The disturbance to projects’ cash flow may turn into insolvency, and eventually business failure. The United Kingdom government tried to solve the problem of late payments through Project Bank Accounts (PBA). The objective of PBAs is to save the cash flow of construction supply chain members from delayed payments. However, the basis for payments between project parties is the construction contract. It imposes how the works are valued and how parties are compensated^21^. Good management of construction contracts is the key to solving problems related to project payments. An endeavor to enhance the contract management process is the use of smart contracts^22^. To implement smart contracts, contract clauses, and subclauses should be turned into structured data forms to be readable by computer protocols. This will introduce a higher degree of accuracy than traditional plain-text contract clauses^21^. However, expert judgment is still required for determinations and other roles in traditional contracts^22^. Mason^23^ suggested that semi-automation of traditional construction contracts will be more appropriate in the meantime since smart contracts interpreting New Engineering Contract (NEC) clauses related to adverse weather conditions, for example, can be achieved. Such a contract will take its input from meteorological recordings, compare it to pre-set criteria, and finally, give an extension of time to the contractor as a remedy for the adverse weather conditions^23^. The use of smart contracts and blockchain in the construction industry enables the automation of making payments and transferring assets between parties and reduce the cost and time of disputes arising from delayed payments and brings down the risks associated with cash flow problems^24^, in addition, the use of smart contracts technology in issuing payments has been praised concerning the security of payments^25^. On the contrary, Turk and Klinc^25^ indicated that the construction industry could be more decentralized using blockchain technology, and more recent efforts are required in this area. Mason^26^ indicated that the semi-automation of a construction contract may be more convenient, especially because the construction industry is slower in adopting technological advancements than other industries. While Li et al.^27^ stated that a mix of traditional and smart contracts will be used in the future.

The emerging blockchain technology is approached for solving issues impeding the collaboration between construction project parties, especially the trust issue. The characteristics of the blockchain technology as transparent ledger, which is immutable, pseudonymous, and resilient are what makes blockchain suitable for solving such problems of the construction industry. With its traceability and auditability, blockchain may strongly change the way businesses are managed in the construction industry. However, the impact of blockchain cannot be witnessed without several steps of testing and validation. The legal aspects and regulations are on top of the list, then the urging need for explicit contract terms and conditions, and finally the mechanisms adopted by contract parties to mitigate risks throughout the lifecycle of a construction project when it comes to disputes. Blockchain technology is suitable in the above-mentioned industry issues where synchronized data and record-keeping of the taken actions are the core values of the blockchain technology^5,27,28^.

The traditional way of data storing in the construction industry follows centrality manner in which all the data are stored in one database accessible from different places. When this database is hacked or corrupted, the main data repository is lost. When the database includes data about money and payments, matters are made worse, especially when payment transaction are made through these databases. The matter is different when using blockchain technology. Since the data is shareable between nodes in the peer-to-peer network, transactions are kept in a block. Once this block is mined, the block is added to the chain with its cryptographic credentials and timestamps that makes the transactions verifiable by the public using the blockchain and so not vulnerable to hacks. Unlike the ordinary internet which is based on moving data from one point to another, the blockchain moves information to the whole system in a synchronized fashion, so nobody has more information than any other body, including the sender himself^4^. Turk and Klinc^25^ indicated that the construction industry could be more decentralized using blockchain technology and more recent efforts are required in this area.

The applications of blockchain technologies could fall in one of the following three categories:

1.Fraud detection and documentation applications in which the burden of keeping the integrity between business operations and imposed regulations is overwhelming and consumes lots of resources. Lots of time is wasted keeping and retrieving data and verifying the originality of documents. With the adoption of blockchain, documents are stored in the blockchain with their history of addition, updating, or deletion is visible to all the nodes, which makes the authentication easier, faster, and more efficient. This category of applications is suitable for data keeping of quality management aspects. For example, data related to the quality of materials, periodic progress reports of construction on site, and concrete, steel and equipment consumption data throughout the construction of a project.

2.Origin and traceability applications in which transparency is of most importance. These applications use the characteristic of transparency in the blockchain as all transactions are visible to all parties. Then tracing back to the root of the transaction to figure out the supply of each item in the project supply chain from quality assurance point of view. These applications are to be of great importance to global projects where for instance, in the case of defects in a part of the project during the operations phase, the history of this part, from manufacturing to transportation, storage, construction and final testing before operations, is kept on the blockchain with their responsible parties known and their input is timestamped which shows easier way of proving responsibilities and avoiding prolonged negotiations of who is responsible. As an example, a blockchain-based model for tracing the supply chain of pre-cast concrete was developed, the model enhanced the information sharing, traceability and the control of scheduling^7^.

3.Payment transactions applications in which the transfer of ownership can be done easily between different parties. The use of such applications in the construction industry enables the automation of making payments and transfer assets between parties and reduce cost and time of disputes arising from payments and equipment leasing.

The use of blockchain technology is sought as a solution that can reduce project disputes arising from delayed payments and bring down the risks associated with cashflow problems^13^.

Weisheng et al.^29^ explored blockchain technology in the governmental supervision of construction work (GSCW). A flexible blockchain-based model was proposed for GSCW with a less degree of decentralization to suit the legislative restrictions of GSCW. Han et al.^24^ reviewed the literature on both Construction Supply Chain (CSC) and Blockchain applications. Their work revealed that CSC issues are related to sustainability, collaboration, and information sharing. The researchers proposed a CSC management system supported by blockchain which bridges the gaps in both fields and enhances the situation of the said CSC issues.

## Framework development

**Figure 1:**
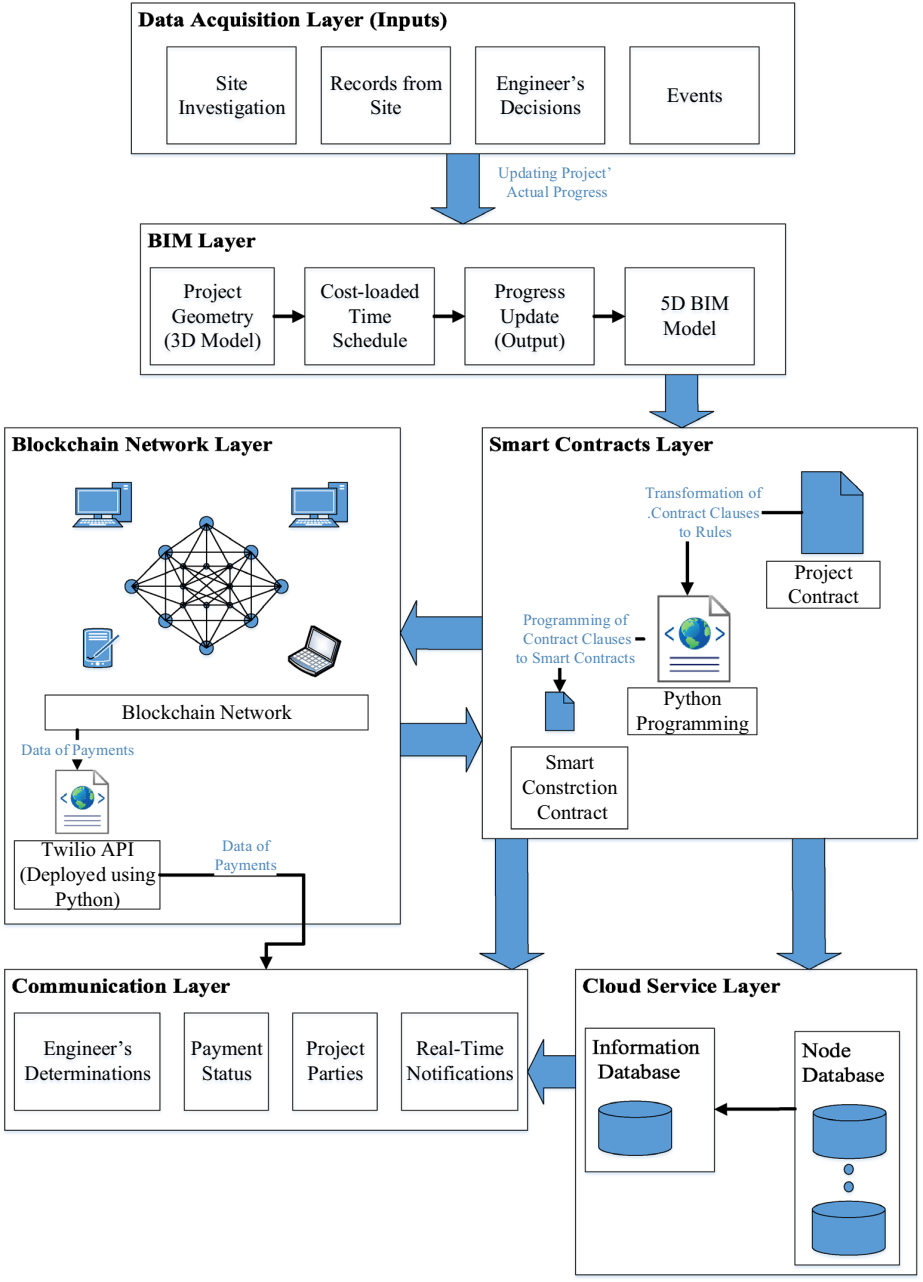
The proposed framework for smart contract development.

The framework adopts a new concept of automating construction contracts using different technologies such as BIM, blockchain, smart contracts, and instant message transfer using the *WhatsApp* mobile application. The framework facilitates making payments from the employer to the contractor in real-time. It also utilizes the functions of the used communication tool in keeping a record of the shared data in chronological order. As the framework is composed of different components, it is divided into interrelated layers as illustrated in Fig. 1.

**Figure 2:**
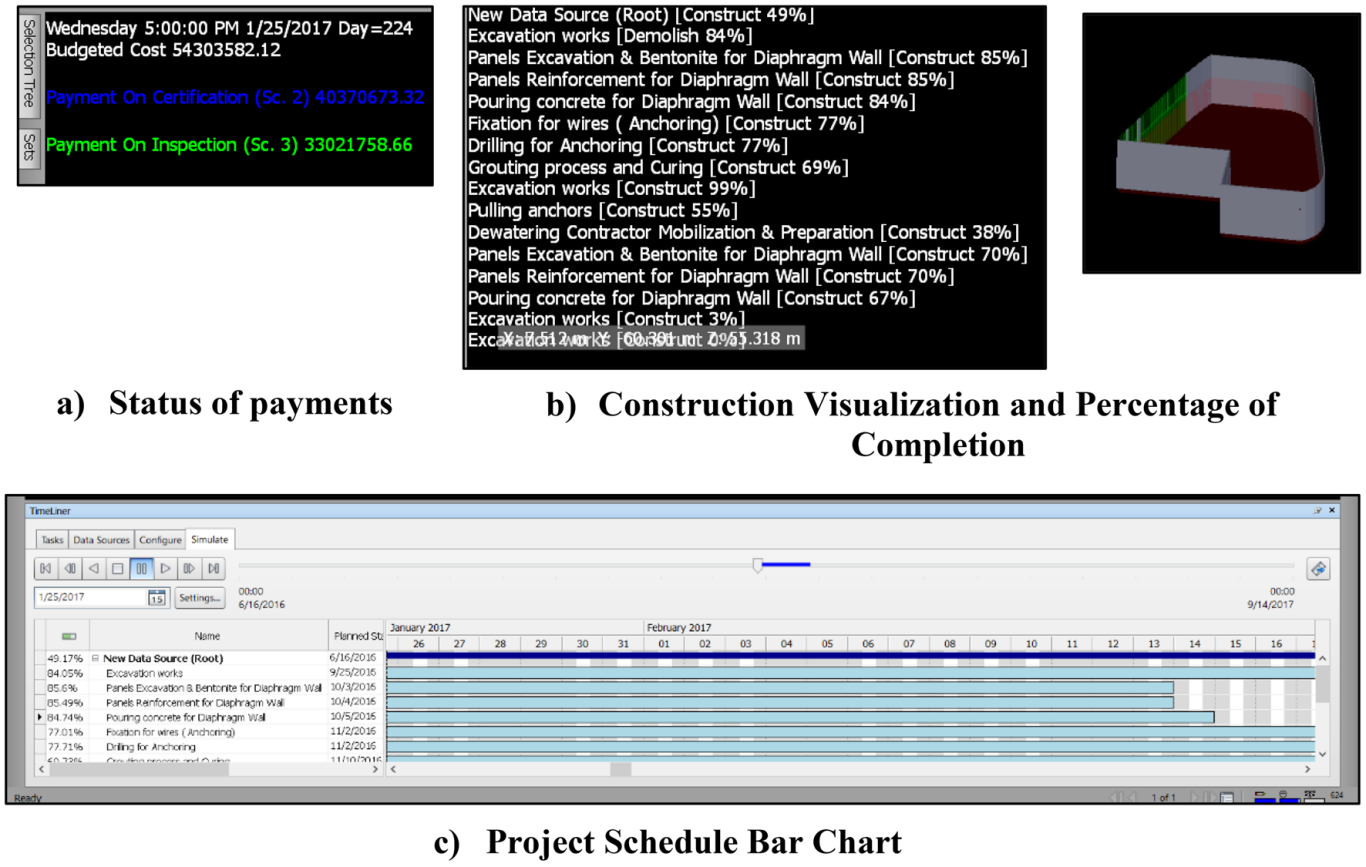
AAA 5D BIM model simulation.

- The framework starts with **Data Acquisition Layer** that collects data from the site. This layer is crucial for the framework since it is the source of actual work executed, and hence the basis for payments. It includes all the data coming from human sources, such as records of work performed and the engineer’s decision about the real work executed.
- **BIM Layer:** 5D BIM model is used for visualizing the project construction timeline, as well as the incurred costs of construction. It considered showing the progress payments of a project, not only its incurred costs. It is important to figure out the status of the project’s cash-in compared to the project’s cash-out. This visualization shall showcase the benefits of the framework as to what extent each of the modeled scenarios has the potential to enhance the project cash flow. The inputs for this layer are project drawings, baseline, and progress updates from the “Data Acquisition Layer”, then the updated 5D BIM model is produced and used for the framework purposes. (See Fig. 2).
- **Smart Contracts Layer:** Since there are dedicated clauses for payments in construction contracts, these terms are used to automate the process of payments upon fulfilling the conditions of these payments’ rules. Smart construction contracts are triggered when the engineer certifies the work executed in real life. Based on the engineer’s update, the amounts due to the contractor are transferred from the updated schedule to the Blockchain Network Layer.
- **Blockchain Network Layer:** this is where project payments are issued. The use of blockchain in this research proposes a new medium for the payment of project invoices without any need for a bank to validate the transactions. Whenever the employer receives the engineer’s certificate, the payment is issued to the contractor. The value and timing of payments are obtained from the smart contracts layer. Afterward, the value of the payment’s transaction and the transaction hash are transferred from the blockchain layer to the communication layer. The transfer of data between the blockchain layer and the communication layer is done through Twilio API.
- **Communications Layer:** It is responsible for communicating data between project parties. The details of construction projects such as the engineer’s and project parties’ decisions, and payment status need to be bundled and communicated together to all project parties.
- To communicate data between project parties, data decentralization is required for a streamlined process. Parties need to get the information in real-time and to be able to check the records and plans on the spot. That is why a **Cloud Service Layer** is a must for completing the objectives of the research’s proposed framework.

### Smart contracts module

Since smart contract applications in literature and real life have proved applicable and efficient, written clauses are interpreted into states and variables using a programming language. This module takes its input from the BIM module-Progress of work and issues the payments accordingly.

### Transformation of contract clauses

For this research application, only remeasured contracts are considered. The obligations and workflow under remeasured contracts are stated clearly in construction contracts with the timing and procedure to be followed. However, some problems still exist. Although conditions of payments are accounted for in construction contracts, payments tend to be delayed.

**Figure 3:**
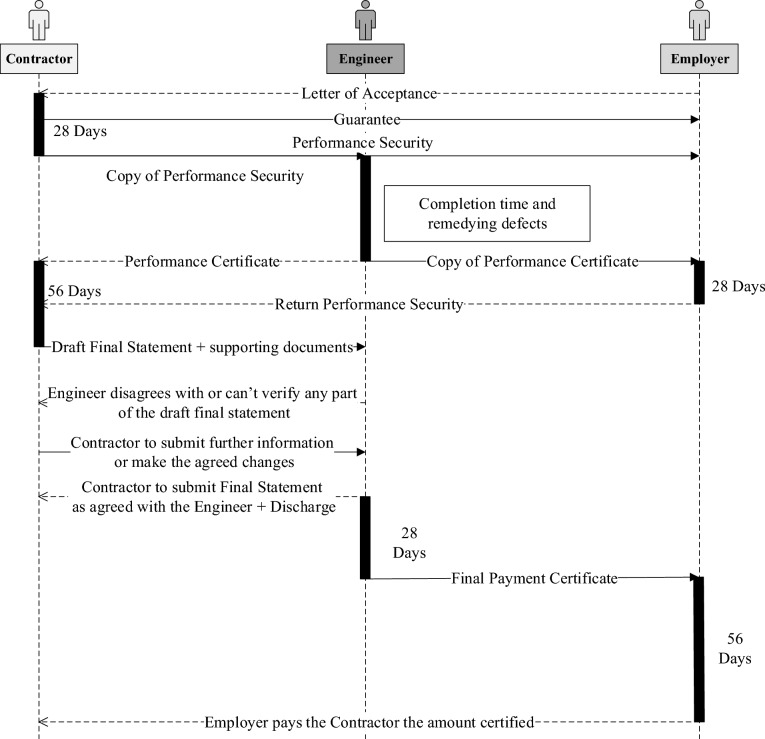
Sequence diagram for FIDIC sub-clause 14.11 application for final payment certificate.

The use of smart contracts along with blockchain has the potential to eliminate the problem of delayed payments. For this reason, contract clauses are transformed into smart contracts, that can be run automatically on the blockchain. This paradigm could save a lot of time and paperwork. It is believed by the literature that human intervention by the Engineer is essential. However, some of his repetitive tasks could be replaced by machine-readable code. For example, the task of certification of contractor’s statements could be achieved Fig. 3.

Smart contracts facilitate the automatic execution of contract terms regarding the issuance of payments based on meeting the conditions stipulated in the construction contract. Among the contract conditions to be met is the actual progress of work, which is obtained from the BIM Layer. The input from the BIM Layer includes the quantities executed and the Engineer’s decision since it is the responsibility of the engineer to validate the work progress reported in the contractor’s monthly statements. the framework supposes that the status of work validated by the Engineer in the updated schedule is the actual work done, and accordingly the contractor is to be entitled to the amounts corresponding to the work done. For this research, the contract payment-related clauses only are translated into smart contracts.

### Programming of contract clauses

**Figure 4:**
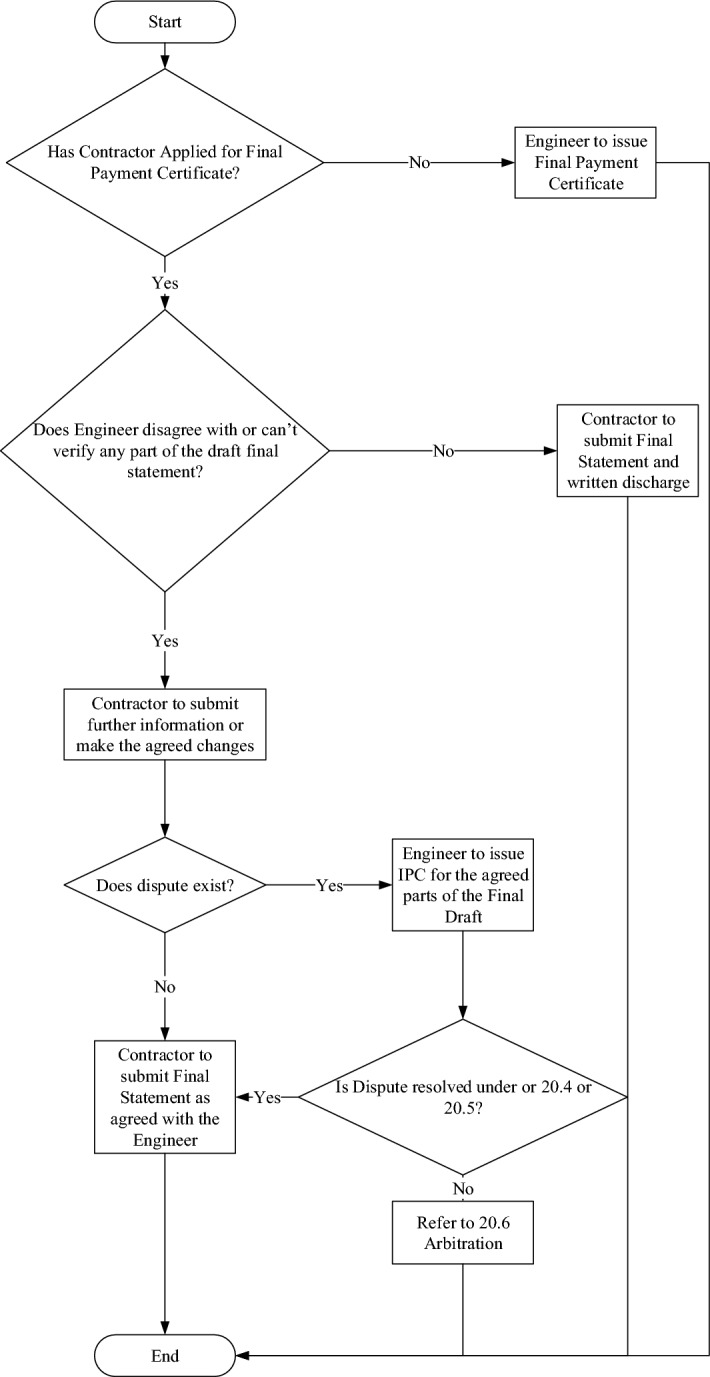
Procedure for FIDIC sub-clause 14.11 application for final payment certificate.

Transformation of contract clauses has been demonstrated to shift the contract paper-based clauses to sequence diagrams that aid computer coding. This section illustrates the coding of clauses using flowcharts. Flowcharts are formed to interpret the inputs, relationships, logical conditions, and outputs of each coded clause. This step is crucial for coding contract conditions (see Fig. 4).

### Blockchain module: construction industry payment scheme

**Figure 5:**
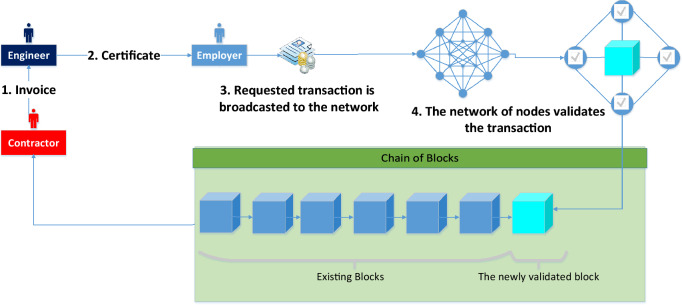
Workflow of the proposed blockchain module.

The module for automated payments is developed using Blockchain technology and smart contracts. Following the traditional way of invoicing, certifying, and paying, the same steps are employed using Ethereum blockchain as shown in Fig. 5.

To develop the framework, *Python* programming language is used, together with two Application Programming Interfaces (APIs) that allow for joining, receiving, and sending transactions on blockchains. Ethereum blockchain has its cryptocurrency. It is a permissionless blockchain, which is accessible to all the blockchain nodes. It supports the use of smart contracts, which means that the blockchain could be programmed. Python programming language is used as it can consolidate nearly all the required operations of the proposed framework. Web3.py library is used. The library can interact with the Ethereum blockchain to help send transactions between Ethereum nodes. Ganache API is used as a personal blockchain that helps developers create and test their applications that run on blockchains, without losing their money^30^. The framework uses Twilio API for *WhatsApp* messaging and is linked to the project’s Excel workbooks.

### Parties’ accounts

To exchange payments between project parties, accounts on the Ethereum Blockchain must be created first. Using the Ganache API, virtual accounts on Ethereum can be created. Each account is created with a unique pair of keys, a public key, and a private key. Both keys are cryptographic codes that are used for ensuring the security of the crypto economy. The public key is used for identification and is public to all the nodes in the Blockchain. To make Transactions between two different accounts, the sender must use his private key to authenticate the transaction and must have the receiver’s public key to send him the amount of money.

### Payments automation

To issue payments on the Ethereum blockchain, the Ganache API is used. The value of a payment is linked to the updated Primavera.XER file to Ganache API. The value of payments is the amounts approved by the engineer in the schedule progress update. The framework is based on validating the contractor’s monthly statement by the engineer and updating the cost-loaded schedule accordingly with the progress of work. The updated.XER file with the monetary amount corresponding to the actual work executed is the value of the payment to be paid to the contractor. The following steps are followed:

1. The schedule.XER file is updated with site progress data.
2. The XER file is saved. XLSX format to be operable with *Python* libraries.
3. The value of each payment transaction is extracted from the. XLSX file to *Python*.
4. Ganache API is called *Python* to be able to issue transactions between project parties on the Ethereum blockchain.

This process of payments automation is followed to remove the need for a third party acting as a validator, such as banks. the traditional payment scheme imposes that a money transfer between two parties is validated by a third party, that is when party A is issuing a payment to party B of any amount, this transaction is made through the bank. This process adds extra costs to the bill as administrative fees.

The computer code reads from the schedule file, then the amount read is issued on the blockchain. Payment Security is an adherent function in blockchain transfers due to blockchain hashing. Because of the blockchain nature, transactions cannot be reversed, and hence money is saved. Using blockchains allows validation of transactions to be reached by cryptographic functions that are running 24/7 for a fraction amount of currency to the node that accomplishes the creation of a new block. The fees associated with transactions are incomparable with bank fees.

To illustrate the cost savings due to using blockchain in project payments, a case study of a keystone retaining wall project in Cairo, Egypt that has 5 months duration and an EGP 29 million contract amount is worked out. EGP as an abbreviation stands for the currency of Egypt (the Egyptian Pound). While ETH as an abbreviation stands for the currency of the decentralized blockchain (Ethereum). It demonstrates blockchain’s transaction costs compared to Commercial International Bank’s (CIB’s) administrative fees and the effect of savings on the contract price. The transaction cost of transferring money from the company’s account to each labor account is 3 EGP. The total transactions carried out along the project for transferring salaries to employees is in the figure of EGP 6,444. While the issuance and administrative fees of debit cards are EGP 45 (paid once for each card), the total card fee is EGP 45,090. The administrative fees for transferring money to vendors and equipment subcontractors are EGP 1400. On the other hand, 1 ETH is divisible by 1 billion Gwei, and a Gwei is divisible by 1 billion Wei. Since the use of ETH is proposed as an alternative currency for the project payments, the exchange rate of ETH to EGP on August 5th, 2020, is taken as an example with the figure of EGP 6343.06.

To make a transaction in ETH sending money from one account to another, some computational work is required for solving the cryptographic problems imposed by the blockchain. Such computational powers are measured in a quantity called gas. The amount of gas consumed is 21,000 gas units for this kind of transaction. With a gas unit price of 1 Gwei, the Ethereum transaction fees cost only EGP 0.13 compared to EGP 3 in traditional banking transactions. Finally, the transaction costs for the considered 5-month project are compared using the traditional banking scheme vs. the proposed Ethereum-based scheme. While there is a significant difference in costs from EGP 52,934 transaction costs using the traditional banking system to EGP 281 using the Ethereum blockchain. The savings due to the proposed scheme is of the figure EGP 52,652, which leads to 0.18% savings to the contract price only from site-related transactions. It is worth noting that general overheads were not taken into consideration. To think about the saving potential for the blockchain as a new financial system, its incorporation in the whole supply chain of construction projects may save more money for project parties. Site-based payments were discussed with saving only 0.18% of the budget of the project, however, in a construction project lots of transactions are made, from insurance to taxes, payments to head office employees, issuance of bonds, and so on. These 0.18% savings could sum up to 0.5%. Considering a margin of profit of 4% for example, these savings lead to 4.5% profit or maybe more depending on the level of adoption of blockchains as a new payment channel. The benefits of using blockchain vs. traditional financial schemes are cost savings of bank fees and instant payments.

### Communication features

**Figure 6:**
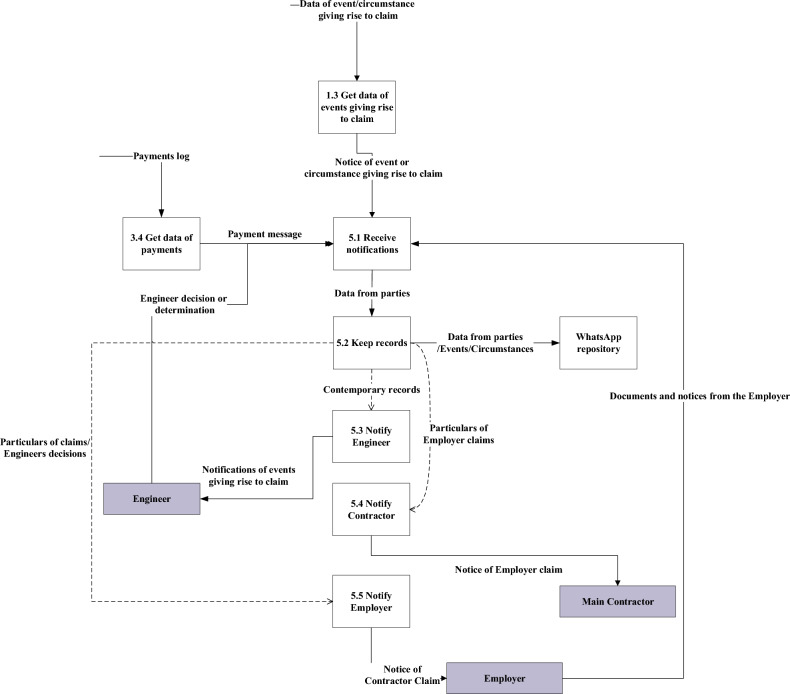
Data flow diagram for communication application.

Communication and the flow of data between project parties are crucial for a successful construction project. The framework proposes a new way of communication that is integrated with the smart construction contract and its associated documents. The framework does not require project parties to extract and communicate important data from scattered project documents. The process of informing project parties of contract updates is done automatically. Using the *WhatsApp* application embedded in the coded smart contract is the solution. Figure 6 shows the data flow diagram of the proposed communication application. The diagram mimics the communication of construction project correspondence between parties. The proposed communication application’s objective is to give project parties real-time notifications of project status and payments.

### Communication application

Messages between parties are sent through the smart contract itself. That is, upon certain conditions or actions carried out by the smart contract, the details of the action are broadcasted to project parties’ WhatsApp accounts. The flow of data in the proposed framework could start with any of the contracting parties as shown in Fig. 6. The Engineer’s role to make decisions or determine the value of work executed or one of the party's entitlements under the contract could be communicated using the communication application as listed in Table 1.

**Table 1:** Communication process for Engineer’s decisions.

Communication aspect	Description	Process ID
Engineer’s Decision	Receiving notifications of the Engineer’s decisions or determinations	5.1
	Keeping a record of the Engineer’s decisions or determinations in *WhatsApp* account memory	5.2
	Notifying the Employer and/or the Contractor of the decision or determination	5.3
Status of Project Payments	Receiving the data of payments form the blockchain	3.4
	Keeping a record of the payment data in the *WhatsApp* account memory	5.2
	Notifying the parties of the status of project payments	5.3
Contractor’s Claims	Getting data from the site of events giving rise to the claim or any other circumstances	1.3
	Receiving this notification of the event	5.1
	Keeping a record of this notification in *WhatsApp* account memory	5.2
	Notifying the Engineer of this claim	5.3
Employer’s Claims	Receiving notifications of Employer’s claim	5.1
	Keeping a record of this notification in *WhatsApp* account memory	5.2
	Notifying the Contractor of this claim	5.3

The communication application receives the Engineer’s decision or determination, keeps a record of these decisions or determinations in *WhatsApp* account memory, and eventually reduces paperwork. And finally, notify the parties of the Engineer’s decision. The status of project payments is kept in the Blockchain. However, they can be communicated in real-time between all project parties. Payments’ data are received from the Blockchain, records of payments are kept in *WhatsApp* account memory, and finally, parties are notified of the payment status. Whenever there is an event where the Contractor considers himself to be entitled to an extension of time or money under the contract, he can notify the Engineer with his intention to claim in real time using the *WhatsApp* application. The Contractor is to get the data of the event or circumstance. Then he sends all data available in addition to his intention of making a claim to the Engineer. All the messages between the Contractor and the Engineer are to be kept in the *WhatsApp* account memory. This procedure provides real-time notification and reduces the probability of missing the claim’s notification period. In case of claims raised by the Employer. The notification of the Employer’s claim is to be received and kept in the *WhatsApp* account memory. Then a notification of the claim is to be sent to the Contractor.

### Cloud service module

The use of cloud service in this framework is for storing and retrieving project data. Site information is to be saved in a project’s drive. This approach enhances the flow of data between project parties and reduces the risk of data centralization. Each node in the network includes some data that is relevant to the node’s owner role, as well as data shared between project parties. For the contractor’s nodes, the type of data stored is more relevant to the execution of the works or anything related to the site. However, this data needs to be communicated with the engineer and the employer in some cases. For example, to study an invoice and issue a certificate by the engineer, the engineer must have access first to all the data about the works executed, the materials on site, bills, and vouchers, or any other piece of data to be used as the basis for the determination. The efficient communication and record-keeping of site data are the basis for saving the contractual rights of parties. Another use of this module is as a data repository that is easily redirected to when using the *WhatsApp* applications in real-time notifications. Using cloud services in this communication endeavor allows for uploading any type of file, sharing its URL with those in charge, and using it to support notifications when used with the *WhatsApp* communication application. The same approach works with conforming drawings with execution. As an example, when setting out a footing on the site, the position of the footing was pre-occupied by an existing building. This notes something that went wrong with the surveying team when mapping the parcel at the beginning of the project. For such problems, the employer is liable not the contractor.

## Case study

To validate the developed smart system, a case study on a construction project is worked out. Several aspects are demonstrated including, (1) project contract is studied for transformation, (2) payments are simulated in the Ethereum blockchain, (3) geometry is represented in a BIM model that is linked to the status of project progress, and (4) the status of payments for each of the framework’s scenarios. The presented case study is for a construction project in Cairo, Egypt. The scope of the work is executing the civil works for a hospital with a built-up area of 7000 m^2^. The project parties are the employer, the design consultant, the main contractor, and the supervision consultant hereinafter referred to as the Engineer. The project budget is 400 million Egyptian Pounds, and the project duration is 18 months. The standard form of contract used is FIDIC Red Book, 5th edition. The study period of the project is from August 1st, 2016, to March 1st, 2018. The data collected in this study are the employer contractor’s contract, drawings, and schedule. The case study contract is visualized and transformed.

### Contract visualization and transformation

**Figure 7:**
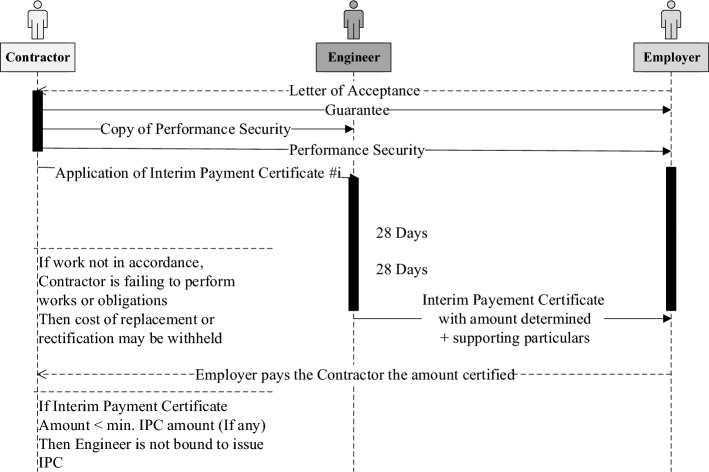
Sequence diagram for FIDIC sub-clause 14.7 monthly statements.

As an example, sub-clause 14.7 of monthly statements are transformed. After the project commences, the Contractor shall prepare monthly statements of the amounts he considers himself to be entitled. This step is to spark the interim payment certification and payment process. Figure 7 shows the process started by the Contractor, preceded by submitting the Guarantee and the Performance Security to the Employer, then the Engineer issues his certificate to the Employer within 28 days of receiving the Contractor’s statement. The Engineer has the right to withhold his certificate if the Contractor fails to perform his obligations, and rectify, or replace part of the works. Or even not issue his certificate if the amount invoiced by the contractor is less than the minimum amount stated in the Appendix to Tender. Once the amount is certified, the Employer shall pay the Contractor within 56 days after Contractor’s statement is received.

### Payments and associated transactions

To adopt the framework in real construction projects, different issues will arise, such as the readiness for shifting from the traditional finance of a project to Blockchain as a new system for payments. The automatic issuance of invoices based on the determined value of executed works by the engineer is another issue. The currency of payment and terms of payment in the construction contract need further amendments to match the potential of a smart construction contract. For these reasons, 3 scenarios are studied for framework adoption. Invoicing as a means of payments in the construction industry is to be disrupted and changed to a more resilient form.

To illustrate the mentioned scenarios, the schedules of payments for each scenario are presented. The amounts of monthly invoices are transferred from the employer to the contractor after the engineer’s certification. Payments are issued on the Ethereum blockchain.

#### Base Scenario and Scenario 1

The base Scenario is the traditional payment scheme of construction projects, starting from monthly statements by the contractor, then the statements are evaluated and certified by the engineer to be finally paid by the employer. Scenario 1 is the same as Base Scenario, except that Scenario 1 uses ETH cryptocurrency for payments. The data of payments are extracted based on the time schedule updates of value earned throughout the project’s execution. As shown in Table 2, the first invoice is submitted in August 2016, and eventually paid in October 2016 as per the contract’s terms. 2 months are essential for studying the invoice by the engineer and issuing the payment by the employer. These scenarios propose no changes to any of the terms of the contract or project circumstances except for the project’s currency of payment which is changed from EGP to ETH for Base Scenario and Scenario 1 respectively. The use of the Ethereum blockchain leads to a reduction in cost and time consumed by banks to issue payments.

**Table 2:** The schedule of invoices and payments in the different Scenarios.

Date	Invoice no.	Invoice value	Payments of invoice		
			Base Scenario and Scenario 1	Scenario 2	Scenario 3
1-Jun-16	–	–	–	–	–
1-Jul-16	–	–	–	–	–
1-Aug-16	Invoice 1	13,771,194.29	–	–	Invoice 1
1-Sep-16	Invoice 2	3,065,217.30	–	Invoice 1	Invoice 2
1-Oct-16	Invoice 3	12,558,324.36	Invoice 1	Invoice 2	Invoice 3
1-Nov-16	–	–	Invoice 2	Invoice 3	–
1-Dec-16	–	–	Invoice 3	–	–
1-Jan-17	Invoice 4	9,904,338.13	–	–	Invoice 4
1-Feb-17	Invoice 5	19,489,233.35	–	Invoice 4	Invoice 5
1-Mar-17	Invoice 6	319,442.91	Invoice 4	Invoice 5	Invoice 6
1-Apr-17	Invoice 7	638,885.82	Invoice 5	Invoice 6	Invoice 7
1-May-17	–	–	Invoice 6	Invoice 7	–
1-Jun-17	–	–	Invoice 7	–	–
1-Jul-17	Invoice 8	8,522,828.50	–	–	Invoice 8
1-Aug-17	Invoice 9	958,858.32	–	Invoice 8	Invoice 9
1-Sep-17	Invoice 10	958,328.73	Invoice 8	Invoice 9	Invoice 10
1-Oct-17	Invoice 11	13,522,674.63	Invoice 9	Invoice 10	Invoice 11
1-Nov-17	–	–	Invoice 10	Invoice 11	–
1-Dec-17	–	–	Invoice 11	–	–

#### Scenario 2

Scenario 2 tends to reduce the period of issuing payments from 2 to 1 month. The rationale behind this proposition is that project parties are aware of their financial arrangements as per the FIDIC contract clause 2.5. This assumption is used to fund the employer’s account with the first payment before commencing the project. Some practitioners may perceive this procedure as not accepted because it limits the employer. However, according to the FIDIC contract, it is a right for the contractor to be aware of the employer’s financial arrangements before commencing the project. Further, the concept of PBA calls for depositing the payments two months in advance. The two months are provided for in the FIDIC red book as the first month is consumed by the engineer to study the invoice and issue the certificate to the employer, and the second month is used by the employer to issue the payment. With clause 2.5 and PBA assumptions in mind, there is no need for the second month to pay the contractor. Table 2 shows the analysis of different scenarios.

#### Scenario 3

Lastly, Scenario 3 assumes payments to be issued on an activity completion basis. That is when an activity is fully or partially completed as per the engineer’s decision, it is approved, certified, and payment is issued instantly. With the use of advanced technologies like computer vision and IoT, this solution could be more applicable.

### Results and discussion

The three proposed scenarios are analyzed through two approaches to figure out the effect of automated payments. Each scenario is analyzed with and without the presence of an advance payment. Finally, the case of real-time payment is analyzed to elaborate its potential to the construction industry payment practices.

#### Approach 1: no advance payment

**Figure 8:**
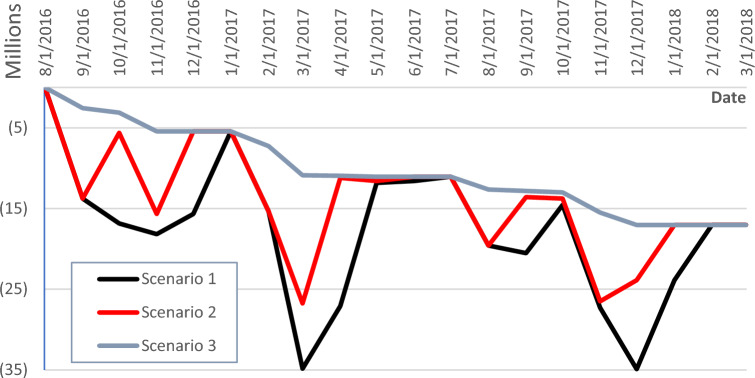
Net cashflow with no advance payment using cryptocurrency-based payment scheme.

This approach assumes no advance payment is provided for in the project contract. As shown in Fig. 8, Scenario 1, an overdraft value of EGP 35,000,000 is recorded on December 1st, 2017. This overdraft value is the worst among all the scenarios as this scenario assumes that payments are released two months after the issuance of the monthly statements, in addition to no advance payments. The cash flow can be enhanced by reducing the payment’s timeframe from two to one month as shown in Fig. 8, Scenario 2. In this case, the overdraft value is reduced from EGP 35,000,000 on December 1st, 2017, to EGP 26,760,000 on March 1st, 2017. This proposition leads to 24% savings in overdraft value, hence reducing the burden on the contractor’s side with no advance payments from the employer. The 30 days required by the engineer as per the contract are maintained, however, the employer is asked to pay the contractor upon the issuance of the engineer’s certificate.

Another alternative is approaching the way of accepting the works and paying the contractor in no time after the issuance of monthly statements. It adds more savings to the contractor’s cash flow by reducing the overdraft value to EGP 17,000,000 on December 1st, 2017, as in Fig. 8, Scenario 3. These 51% savings relieve a big portion of the financial risk of insolvency away from the contractor’s side. However, it adds a burden to the engineer who is responsible for inspecting and approving the works as fast as possible. Although the role of the engineer is now more amplified, the other roles of quantity take-off from drawings, studying bills, vouchers, receipts, etc. are carried out by the proposed framework.

#### Approach 2: advance payment

**Figure 9:**
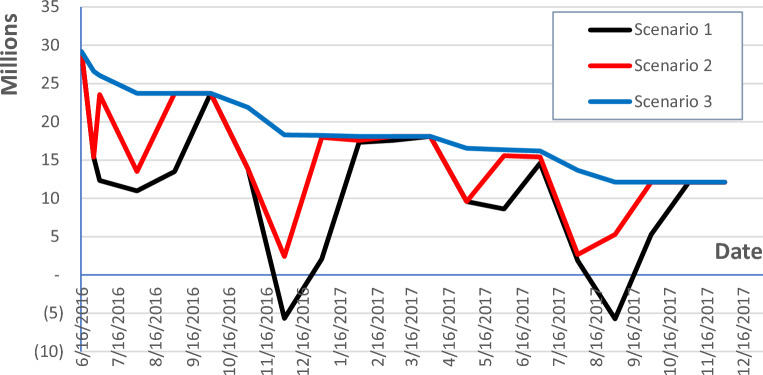
Net cashflow with advance payment using cryptocurrency-based payment scheme.

Figure 9 shows the project’s cash flow after receiving an advance payment of EGP 29,164,000 on August 1st, 2016, as per the contract, the project’s cash flow for Scenario 1 is way more enhanced with a maximum negative value of EGP 5,740,000 on December 1st, 2017. This 84% saving in the overdraft value is incomparable to the contractor taking the previously mentioned alternatives with no advance payment into consideration.

With the augmentation of reducing the payment timeframe from two to one month with the advance payment, no negative value appears in the project’s study period. This means that the contractor is not using his equity when executing the project. As depicted in Fig. 9, Scenario 2 shows the effect of payment on certification on the project’s cash flow, with no overdraft value. By adding an advance payment to the above-mentioned Scenario 3, the proposed cash flow is more flattened and no negative values are experienced as shown in Fig. 9.

#### Real-time payment

**Figure 10:**
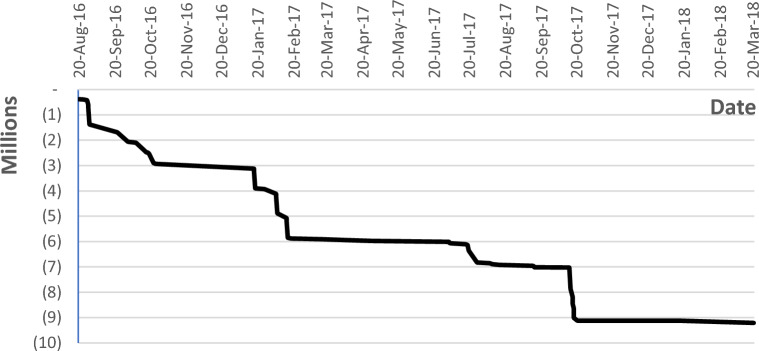
Real-time payments with no advance payment.

This section analyzes the payments to be issued on an activity completion basis. That is when an activity is completed as per the engineer’s decision, it is approved, certified, and payment is issued instantly. With the use of advanced technologies like computer vision and IoT, this solution could be more applicable. The potential for this solution does not only lie in the instant payments and saved cash flow, but it is also about the agility in dealing with each activity in real time. To deal with the quality, cost, and any circumstance that may lead to any delays to the activity completion. Figure 10 shows the effect of this approach on the project’s cash flow with no advance payment and with a maximum overdraft of EGP 9,130,000 in the project study period.

The roles of the engineers working in this area of expertise may see some differences as it is not the way often followed. However, the differences are expected to be the ability to work with the new technology along with their daily tasks. It is about how they perceive the quality of the activity executed and the percentage of completion, then update the framework with the work status. A smart contract is to issue or withhold the payment to the contractor. More engineering work, but nearly no paperwork.

### Communication between project parties

**Figure 11:**
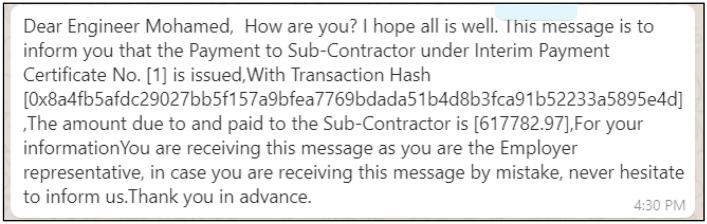
Real-time notifications of payments to subcontractors.

*WhatsApp* application is used for communication between parties. Figure 11 shows an example of correspondence regarding the status of payment to the subcontractor. The message includes the amount paid, the transaction hash, and the reference number on the Ethereum blockchain.

### The bottom line

The research presented a background of the construction industry payment-related issues. It elaborated on some efforts exerted to solve these issues like Project Bank Accounts (PBAs). Then the research reviewed modern technologies that showed potential for solving some of the construction industry’s problems. Among the technologies reviewed were BIM, smart contracts, and blockchain technologies. These technologies have been adopted by different industries and demonstrated high benefits and sustained different businesses across these industries. Construction industry also has been on the way to adopting these technologies after they proved efficient and disruptive to different sectors and industries. The research continued to explore the most famous application of blockchain and smart contracts’ technologies, cryptocurrency. Finally, the research discovered the opportunity for integrating these technologies to develop a smarter construction contract based on the automation of its payment processes.

Notwithstanding the mentioned efforts of researchers in transforming the construction payment scheme to a smarter one, there is a gap in transforming the contract clauses into smart contracts. Nanayakkara et al.^31^ concluded that solutions based on blockchain and smart contract can significantly mitigate the issues of poor payment practices. Others like Sigalov et al.^32^ have indicated that smart contracts are of much value regarding the automation of payments in the construction industry, they further theoretically affirmed the ability of smart contracts and BIM to automate the delivery phase of a project, in addition to automating the issuance of payments by linking the BIM model with the project’s contract. And finally, Li et al.^15^ introduced the use of smart contracts for automating the issuance of payment for a limited installation activity by coupling BIM requirements with smart contracts. The research contribution is the transformation of contract clauses to smart contracts. For the purposes of this research, the said transformation is limited to automation of project payments. This partial transformation of contract conditions is believed to be suitable in the meantime as reported by Mason, et al.^26^, who thought that semi-automation of contract conditions could be the solution in the meantime.”

## Conclusions

The research presented a framework that integrates Blockchain, BIM, and Smart Contracts technologies as a step toward the construction of smart contracts. The research proposed different scenarios for the adoption of a payment automation framework. Firstly, it started with the (Base Scenario) which represented the traditional way project payments are issued. Secondly, it proposed a new currency for project payments which are ETH, (Scenario 1). The use of cryptocurrencies proved efficient in reducing the administrative fees of banks processing different project payments, as well as the speed of issuing payments. Thirdly, it modified the traditional workflow of construction project payments by replacing the accounting role, calculating the deduction amounts in monthly invoices, with smart contracts (Scenario 2). Finally, it proposed changes to the contract management process and project payments be activity-based and not monthly-based (Scenario 3). This proposed solution by Scenario 3 is the result of integrating the different technologies.

A case study of real construction project in Cairo, Egypt was used as a case study. The three scenarios were implemented through the case study. Two approaches were taken; the first approach perceived three alternatives assuming no advance payment, and the second approach assumed an advance payment for the same alternatives. Finally, one last case of activity-based instant payments was analyzed and concluded. The developed framework can assist project teams with contract management. This research can be extended in the future to include different procurement routes like Engineering, procurement, and construction (EPC) contracts. The use of IoT/Computer Vision in the inspection of task completion is a further step toward full automation. This research contributed to the body of knowledge by developing a construction smart contracts framework to assist the management teams with payment-related clauses and cash flow enhancement. It proposed blockchain as an efficient alternative financial system with better cost savings compared to the traditional one used in the construction industry. This research is limited to the role of work inspection and engineering actions to the engineer.

### Future research

This research can be extended in the future by validating the model using CBDCs as a currency of payment after finishing its development stage. The use of CBDCs facilitates the run different businesses. Also, research can be conducted to study the effect of using CBDCs with respect to the different regulations of each issuing central bank needs to be studied carefully. Unlike public cryptocurrencies, CBDCs are governed by their issuing banks, which imposes another layer of legislations to the parties in a construction project.

## Data Availability

All data generated or analyzed during this study are included in this published article.
